# Spatial Variation in Risk for Highly Pathogenic Avian Influenza Subtype H5N6 Viral Infections in South Korea: Poultry Population-Based Case–Control Study

**DOI:** 10.3390/vetsci9030135

**Published:** 2022-03-15

**Authors:** Saleem Ahmad, Kye-Young Koh, Dae-Sung Yoo, Jae-Il Lee

**Affiliations:** 1 Veterinary Public Health Lab, College of Veterinary Medicine, Chonnam National University, Gwangju 61186, Korea; 178510@jnu.ac.kr (K.-Y.K.); jaeil@jnu.ac.kr (J.-I.L.); 2 Animal and Plant Quarantine Agency, Gimcheon 39660, Korea; shanuar@korea.kr

**Keywords:** highly pathogenic avian influenza, hotspots, kernel density estimate, spatial scan statistic, clusters

## Abstract

Given the substantial economic damage caused by the continual circulation of highly pathogenic avian influenza (HPAI) outbreaks since 2003, identifying high-risk locations associated with HPAI infections is essential. In this study, using affected and unaffected poultry farms’ locations during an HPAI H5N6 epidemic in South Korea, we identified places where clusters of HPAI cases were found. Hotspots were defined as regions having clusters of HPAI cases. With the help of the statistical computer program R, a kernel density estimate and a spatial scan statistic were employed for this purpose. A kernel density estimate and detection of significant clusters through a spatial scan statistic both showed that districts in the Chungcheongbuk-do, Jeollabuk-do, and Jeollanam-do provinces are more vulnerable to HPAI outbreaks. Prior to the migration season, high-risk districts should implement particular biosecurity measures. High biosecurity measures, as well as improving the cleanliness of the poultry environment, would undoubtedly aid in the prevention of HPAIV transmission to poultry farms in these high-risk regions of South Korea.

These authors contributed equally to this work.

## 1. Introduction

Since 2003, highly pathogenic avian influenza (HPAI) H5 viruses have circulated across most of the world’s countries, causing massive economic consequences and significant public health concerns. The spread of HPAI H5 has been driven by the migration of wild birds [1]. The HPAI H5 viruses have diversified into several genetic lineages and clades, and these viruses are still evolving. New recombinant HPAI viruses, in clade 2.3.4.4, were found in poultry and wild birds, which remain affected in South Korea since January 2014 [2].

When avian influenza viruses cannot be controlled from spreading across poultry productions and businesses in time, poultry may become a reservoir [3]. HPAI was first reported in Korea during the 2003–2004 growing season, and outbreaks have happened every year since. HPAI epidemics started off minor, but have since grown in size and severity. During the early epidemic, nineteen farms in ten regions were infected. The epidemics expanded across a broader region spanning nineteen regions a few years later, in 2008–2009, when the number of confirmed farms afflicted by the virus had risen to thirty-three in total. HPAI epidemics were comparable in magnitude and scope during the 2014–2015 production season, with 38 farms confirmed afflicted in nineteen regions. A much more significant HPAI outbreak took place in 2016–2017. Between October and November 2016, recombinant clade 2.3.4.4 H5N6 HPAIVs were isolated from migrating birds in South Korea. Following that, HPAI outbreaks in about 380 poultry farms were reported by the ‘Animal and Plant Quarantine Agency’ (APQA) in South Korea by 4 April 2017 (http://www.qia.go.kr accessed on 15 August 2021). H5N6 HPAIVs were detected and identified in China in 2013, and have subsequently expanded not just to China, but also to Vietnam and Laos. The HPAIV subtype H5N6 in South Korea was discovered to be a unique reassortant of H5N6 viruses detected in China and Eurasian low-pathogenicity AI viruses found in wild birds in England, Germany, Japan, and Taiwan. The Ministry of the Environment reported, in 2017, that HPAI epidemics in Korea frequently coincide with seasonal bird migration patterns [4]. Overall, clade 2.3.4.4 H5N6 viruses have propagated quickly and generated widespread epidemics in poultry [5].

The necessity for effective countermeasure applications in high-risk locations has been highlighted by multiple HPAI H5 epidemics in South Korean poultry production systems. Risk analysis of HPAI is critical for ‘Public Health Emergency Preparedness’ (PHEP) in the occurrence of an epidemic, especially in underserved areas in South Korea [6]. In this study we explored methods to understand and predict risk across regions in South Korea from case and control poultry farms. Previous research emphasized the spatiotemporal dynamics of HPAI H5N8 clade 2.3.4.4 in order to identify high-risk zones [6]; however, HPAI H5N6 clade 2.3.4.4 has not been analyzed for spatial clustering to detect high risk locations (hotspots). We analyzed the HPAI H5N6 epidemic in South Korean poultry farms from 2017 to 2018 and identified high-risk areas in this study. In order to identify high risk sites for HPAI H5N6 clade 2.3.4.4, we used kernel density estimation and spatial scan statistics with Ripley’s K function. The geometry of the region being scanned, the probability distribution of producing events under the null hypothesis, and the types and sizes of the scanning window are the three essential features of the scan statistic [7]. The Ripley’s K function is, indeed, a quantitative method used in the study of spatial and spatiotemporal point patterns. The goal of spatial point pattern analysis is to measure the distribution of point occurrences in a two-dimensional geographical area. It does have a wide range of uses, particularly in epidemiology. Ripley’s K function classifies a set of points and differentiates between random, clustered, and regular patterns [8]. Ripley’s K function is a technique for assessing completely mapped spatial point process data, such as data on event locations [9].

This study will identify the clusters of HPAI cases in South Korean regions as hotspots. High levels of attention from public health officials regarding proper measures to minimize risk in the neglected regions will help to prevent avian influenza outbreaks in the future.

## 2. Materials and Methods

Comprehensive information about the case and control poultry farms during the HPAI H5N6 epidemic were extracted from the “Epidemiology report for outbreak of highly pathogenic avian influenza (HPAI) in Republic of Korea”, issued by the South Korean animal and plant quarantine agency (APQA). Since the first HPAIV outbreak at a chicken farm in 2003, the Republic of Korea (ROK) has established a very vigilant monitoring program on poultry farms throughout the country for preventative and intelligent monitoring. For example, all poultry farms require their poultry to be inspected before slaughter or movement (approval of their poultry for transportation). Furthermore, during the HPAI outbreaks, domestic duck farms that normally exhibited no clinical indications were subjected to bi-weekly monitoring tests. Infected poultry often displayed significant clinical symptoms, such as mortality, lower egg production, and a higher daily fatality rate, allowing for early discovery and control of further transmission. Soon after the discovery of poultry with clinical signs of HPAI infection by livestock owners, farm laborers, or veterinarians, the situation had to be disclosed to the Animal and Plant Quarantine Agency (APQA), Gimcheon, Korea, in passive surveillance, as defined by the Law on Prevention of Contagious Animal Diseases. Veterinarians from the government went to the suspected poultry farms to collect samples from sick or dead poultry, which were then tested for HPAI infection [10,11]. If suspected farms samples were positive for HPAIV, the farm in question was deemed an infected premise (IP). Additionally, poultry farms that were epidemiologically related to infected premises, such as through shared vehicles, or were situated at a nearby distance (e.g., 3 km), were subjected to an HPAIV test, followed by pre-emptive depopulation. A depopulated farm that screened positive for HPAIV was recognized as a positive premise in the surveillance [10,11]. In this regard, without any surveillance reporting, it is exceedingly unlikely that the control farms would be infected. The dataset regarding case and control poultry farms is available in the Supplementary Materials (Supplementary File.zip). We applied kernel density estimation and a spatial scan statistic to identify high-risk areas with significant clusters of HPAI outbreak cases [12,13].

The entire analysis was performed with the help of R [14]. Stepwise ‘R code’ or ‘R.Script’ is available for the whole analysis in Supplemental Materials (Supplementary File.zip). Before starting the analysis with R, various packages were installed and loaded into R in the first phase, in order to carry out the analysis successfully. These were: “rgdal” [15], “raster” [16], “ggplot2” [17], “plotrix” [18], “fields” [19], “leaflet” [20], “maptools” [21], “RColorBrewer” [22], “lattice” [23], “geoR” [24], “car” [25], “sp” [26], “spdep” [27], “ape” [28], “pgirmess” [29], “spatstat” [30], “splancs” [31], “smacpod” [32], “Metrics” [33], “gtools” [34], “lme4” [35], “oro.nifti” [36], “tidyverse” [37].

In the 2nd phase, the dataset regarding case and control poultry farms was obtained and arranged in excel 2016 as point process data. The excel file was then converted into a CSV file. The obfuscated data containing cases and controls, along with their longitude and latitude, were prepared before performing the analysis in R. The CSV file was loaded into R. Objects of cases and controls were created. A new object with just the case farms was set as “1”, while an object with just the control farms were set as “0”. In the 3rd phase, the Case–Control data were then created as a “Spatial Points Data Frame”. In the 4th phase, a boundary file for South Korea was downloaded from GADM, the Database of Global Administrative Areas (https://gadm.org/data.html, accessed on 15 August 2021) with the getData function in R. In the 5th phase, the location information for 360 cases and 3206 controls was included in the CSV data. The variable type for cases and controls, which were designated as 0 and 1 in the dataset, was integer. As a result, it was added to the dataset as a factor/categorical variable. Case and control datasets were separated for further analysis. The CSV file was converted into an sf object and cases and controls were visualized using ggplot. In the 6th phase, a kernel density estimate was generated for visualizing the risk on the map. For this purpose, a point pattern object of points (based on x and y cartesian coordinates) was created, with the points denoting the population of cases. To begin, an owin window object that defines the population from which the cases emanated was constructed. In the 7th phase, the cases’ point pattern ‘ppp’ object was specified. In the 8th phase, a kernel density estimate was generated with different bandwidths, and plotted to visualize the intensity of points (cases) per unit square. Increasing the bandwidth smooths the visual of the estimate, while reducing the bandwidth may result in the identification of false zones with greater risk [38]. As a result, the smoothing bandwidth sigma ought to be a single numeric number that represents the standard deviation of the isotropic Gaussian kernel. Furthermore, bandwidth was chosen using cross-validation methods such as “bw.ppl.”. Finally, the density estimate was visualized on the maps using leaflet.

In the 9th phase, using the Kelsall & Diggle method [39], the ratio of the density estimate of cases: controls was calculated. To begin, ‘marks’, simply numerical values assigned to each point such as case or control (1,0) were added to the points. For this purpose, the “relrisk” function in R with a package named “spatstat” was used to assess the risk of being a case, relative to the surrounding population (controls). To achieve a relative risk result, we set relative = TRUE in the R code for the desired output as the probability of being a case, relative to the probability of being a control. Cross-validation was used to find a common bandwidth to use for cases and controls, without setting sigma or bandwidth, in the 10th phase of our analysis.

## 3. Spatial Autocorrelation and Clustering of Point Process Data

In the 11th phase, the ‘cases’ and ‘controls’, after converting the data to a SpatialPolygonDataFrame (SPDF), were plotted and visualized. We looked at first and second order functions, summarizing the spatial dependency between events, after producing a first-degree kernel density estimate and determining the proportion of the density estimate of the case and control groups. For this purpose, we converted the data collected to a “point pattern” PPP data frame in the 12th phase of the analysis. In the 13th phase, the spatial dependency between events was summarized using Ripley’s K function over a wide variety of spatial scales, with the help of the “spatstat” package. The estimate of K(r) was plotted.

In the 14th phase, a confidence envelope using the Monte Carlo (MC) simulation was plotted. Spatial autocorrelation and spatial clustering were performed to check whether the high-risk areas had clusters of HPAI H5N6 epidemic cases. For this purpose, using two methodologies, we investigated the variance in Ripley’s K function between case and control groups.

## 4. Methodology 1

According to Baddeley, A et al. [30], K function vignette basically evaluates the K function for case and control groups and compares the results. First a marked point process was created and the K functions for cases and for controls were calculated. Then the differences in the two functions were calculated in the 15th phase of our analysis.

## 5. Methodology 2

In the 16th phase, the kdplus test was used to perform a global clustering test utilizing differences in K functions [40]. A package called “Smacpod” was used. A function in the “Smacpod” package estimates the difference in the K function and plots a simulated confidence interval “CI”. We finally looked at a spatial scan statistic. The Case–Control object was converted to a “PPP” object before performing the spatial scan statistic. For the spatial scan statistic, we used the “smacpod” library in R to run the Kulldorf spatial scan statistic. The clusters identified with the spatial scan statistic were finally visualized using leaflet in the 17th phase.

## 6. Results and Discussion

**Figure 1:**
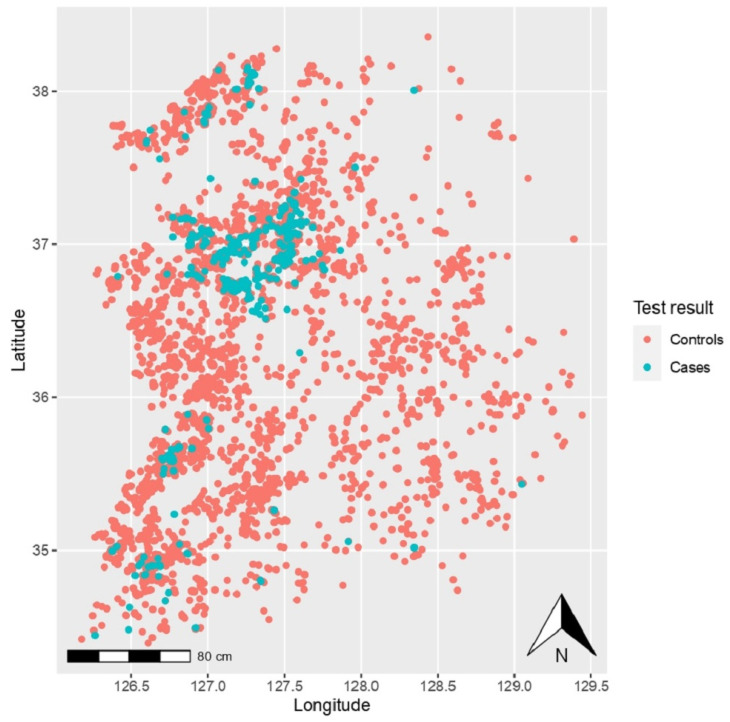
Distribution of highly pathogenic avian influenza cases (denoted by blue dots) and controls (denoted by red dots) across the country (South Korea).

The HPAI H5N6 epidemic in South Korean poultry farms occurred in a single phase, continuously, for 107 days; it lasted from 16 November 2016 to 3 March 2017, and resulted in a total of 343 HPAI H5N6 epidemic cases. The distribution of cases is shown on ggplot to indicate the risk distribution, as illustrated in Figure 1.

**Figure 2:**
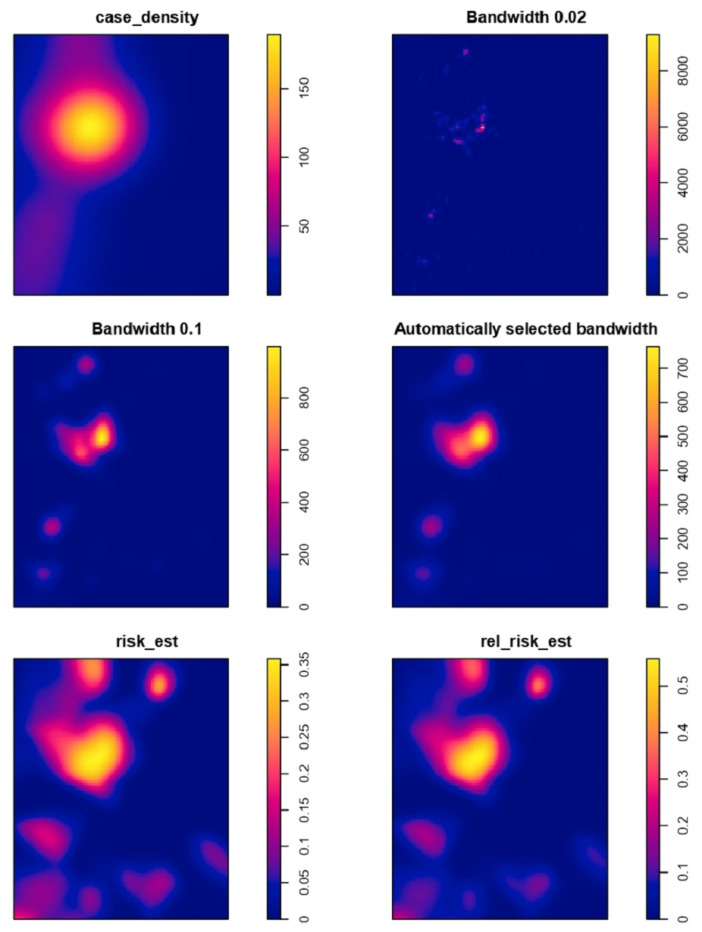
Kernel density plots displaying the density of case farms (**top left**) by kernel density estimation based on multiple bandwidths of 0.02 (**top right**), 0.1 (**middle left**), and automated bandwidth selection by cross validation (**middle right**). Risk estimate (**bottom left**) and relative risk estimate (**bottom right**) with colored scale bar on the right denoting intensity of risk from blue to yellow, with a relative unit of measurement used to represent the proportion of risk value.

Assessing the risk of HPAI H5N6 recurrence associated with previously observed point events is crucial for adopting effective HPAI control management. For Converting HPAI case point data into continuous geographical distribution data, Kernel density estimation (KDE) was used to describe the geographical distribution of HPAI H5N6 outbreaks, and to reduce the effect of previous information in point format with incorrect locations. With the help of a point pattern object based on longitude and latitude cartesian coordinates, kernel density estimate of cases with different bandwidths were generated, which can be observed in Figure 2. A bandwidth that is too high or too low risks over- or under-smoothing the original data. Because future studies rely on estimated density information rather than actual points, a variation in bandwidth may have a great influence on statistical correlations between the dependent and independent variable [41]. Density calculations can be applied to either cases or sites. Cases are occurring spots of disease, while sites are all the points on a grid in a study area. Density calculations applied on sites using a site-side approach evaluate density for all the locations in the study area, while the case-side method solely considers case locations and their specified nearby points [41].

Kernel density estimation (KDE) is a spatial analysis approach that takes into consideration the relative positions of objects [42]. To gain a better understanding of the high-risk population, visual representation may be used to comprehend the population at risk due to a larger number of HPAI cases occurring in that location instead of an actual increase in risk. Therefore, rates were estimated using the Kelsall and Diggle approach (a cross validation method), allowing kernel estimates for relative risk to be produced.

**Figure 3:**
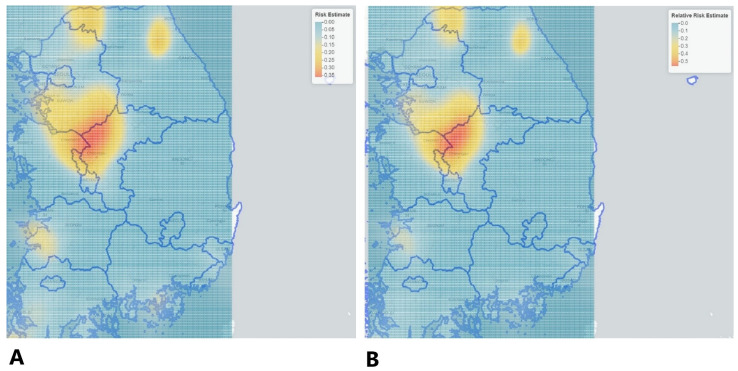
Depicts high risk locations on the map of South Korea (Ballatore, Bertolotto & Wilson, 2013). Kernel density risk estimate (**A**) and relative risk estimate (**B**) of being a case relative to being a control. Note: Chungcheongbuk-do, Chungcheongnam-do, Gyeonggi-do, and Jeollabuk-do provinces showed high risk of HPAI H5N6 epidemic.

The intensity of risk relative to the control is depicted in Figure 2 and Figure 3, respectively. According to the relative risk estimate output, the relative risk of being a case (affected location) vs. being a control (non-affected location) in our HPAI H5N6 case–control data varied from 0 to 0.053 (0% to 5%), which can be seen in Figure 3 and in the relative risk estimate in the CSV file available in the Supplementary Materials. The risk estimate and the relative risk can be observed on the leaflet (map) depicted in Figure 3.

**Figure 4:**
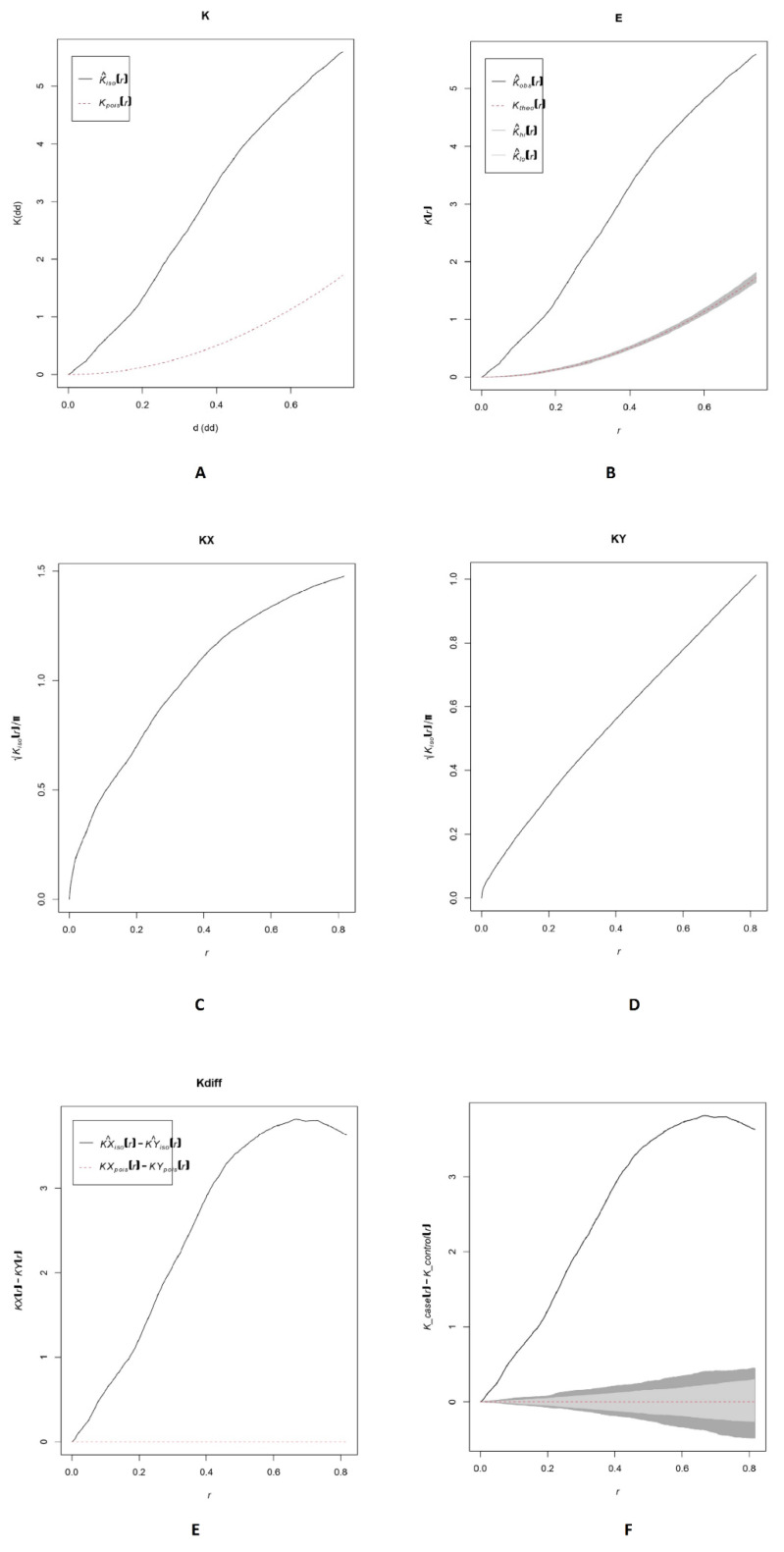
Ripley’s K function for point process pattern and deviation from complete spatial randomness. Panel (**A**) shows that the actual measured value of K is higher than the theoretically determined anticipated value at all ranges tested, demonstrating significant clustering. Panel (**B**) shows the grey zone as a randomization envelope. Across all distances, actual observed values (K) are much higher than the envelope of predicted K values, indicating significant clustering and divergence from complete spatial randomness. Panel (**C**) shows the actual observed values for cases. Panel (**D**) shows the actual observed values for controls. Panel (**E**) shows differences in actual observed values for cases and actual observed values for controls. Panel (**F**) shows “difference in K function”, denoting that actual observed value of K function is, again, above the grey zone of randomization. Dark grey is minimum/maximum while light grey is the confidence envelope indicating deviation from complete spatial randomness.

In our point pattern analysis, it is critical to determine if a point distribution has a a clustered or dispersed pattern by employing a quantitative measure that reflects the extent of clustering. Point pattern analysis is one of the fundamental approaches in spatial analysis, dealing with the distribution of homogeneous points, that is, one type of point, focusing solely on the spatial element of point distributions and avoiding their characteristics. Complete Spatial Randomness (CSR) defines the absence of any pattern in the spatial points [9]. According to the output of Ripley’s K function, considerable clustering was identified at practically all distances showing deviation from CSR, as illustrated in Figure 4 for spatial dependency (K-plot).

**Figure 5:**
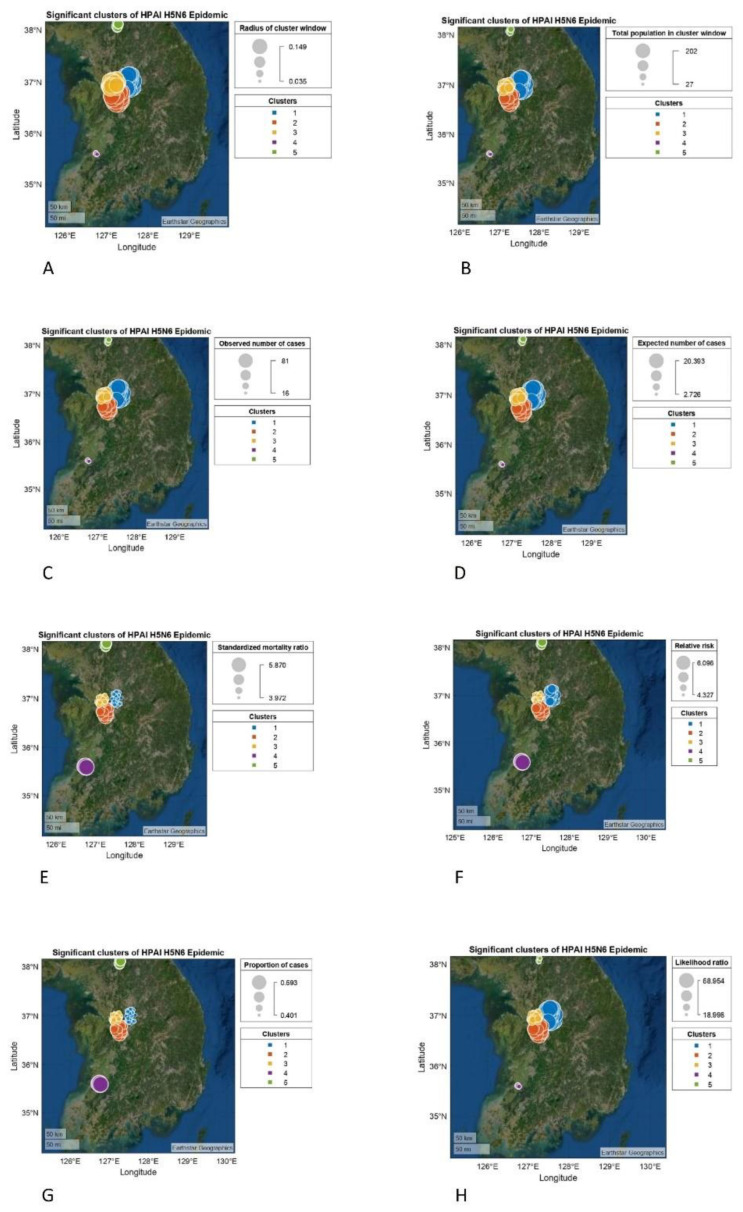
Spatial clusters for the highly pathogenic avian influenza H5N6 epidemic (represented by different colors) detected in the provinces of Chungcheongbuk-do, Chungcheongnam-do, Jeollabuk-do and Gyeonggi-do: (**A**) radius of cluster window showing that clusters 1, 2, and 3 were, respectively, higher than cluster 4 and 5; (**B**) total population (number of poultry farms) was higher in cluster 1 followed by clusters 2, 3, 4, and 5; (**C**) showing that the observed number of cases was highest in cluster 1, followed by clusters 2, 3, 4, and 5, respectively; (**D**) showing the expected number of cases in all clusters; (**E**) showing that the standardized mortality ratio was highest in cluster 4, followed by clusters 5, 2, 3, and 1; (**F**) showing relative risk in all significant clusters. Relative risk of cases was higher in cluster 4, followed by clusters 5, 2, 1, and 3; (**G**) showing the highest proportion of cases in cluster 4, followed by clusters 5, 2, 3 and 1; (**H**) showing that the likelihood ratio was highest in cluster 1, followed by cluster 2, and clusters 3, 4 and 5. See Table 1.

Moreover, the significance level of the pointwise Monte Carlo test was recorded as statistically significant (*p* = 0.002). The Diggle and Chetwynd (1991) test for difference in K functions [43] was also recorded to be statistically significant (*p* = 0.001, KD = 12578.13). Finally, five significant clusters were detected during the H5N6 epidemic as a result of the analysis of spatial scan statistics, which is depicted in Figure 5.

During the HPAI H5N6 outbreak, five clusters were identified by spatial scan statistic. The center of cluster 1 was located at “307 Sinpyeong-ri, Geumwang-eup, Eumseong, Chungcheongbuk-do”, comprising 202 locations in its radius. There was a total of 81 cases identified in cluster 1. The center of cluster 2 was detected at “746 Bongyang-ri, Seongnam-myeon, Dongnam-gu, Cheonan, Chungcheongnam-do” and its radius covered 104 regions. Cluster 2 was found to be significantly related with 46 cases in total. The center of cluster 3 was detected at “46 Husaengchon 2-gil, Seonghwan-eup, Seobuk-gu, Cheonan, Chungcheongnam-do”, and covered 76 locations in its neighborhood. There were 31 cases that were substantially linked to cluster 3.

The center of cluster 4 was found at “440-20 Sinjung-ri, Gobu-myeon, Jeongeup, Jeollabuk-do”, and its radius comprised 27 locations. There were 16 cases found in total. The center of cluster 5 was detected at “152-15 Yeongbuk-ro, 238 beon-gil, Yeongbuk-myeon, Pocheon-si, Gyeonggi-do”; its radius covered 43 locations, and 20 cases were linked to cluster 5. The location IDs (geographical coordinates) of all the poultry farms associated with clusters 1, 2, 3, 4 and 5 are listed, respectively; they are available in the Supplementary Materials (See Supplementary File).

The kernel density estimate and spatial scan statistic both identified districts of the Chungcheongbuk-do, Chungcheongnam-do, Gyeonggi-do, and Jeollabuk-do provinces to be higher at risk of HPAI H5N6 outbreaks. The Chungcheongbuk-do, Chungcheongnam-do, Gyeonggi-do, Jeollabuk-do provinces had high domestic chicken and domestic duck poultry density (number per km) [44]. According to Kwon et al. [45] a higher density of domestic ducks might lead to a higher number of avian influenza outbreaks. The viral transfers from wild birds to domestic ducks and domestic ducks to chickens were particularly essential. Domestic ducks in South Korea are more vulnerable to an HPAIV invasion than domestic chickens. The primary carriers for avian influenza viruses are waterfowl [46]. According to the literature, ducks may have a role in the regulation of HPAI (H5N1) viruses. Although infected ducks may show no symptoms, they can excrete significant levels of the virus, which is detrimental to other species of poultry [47,48,49,50]. Moreover, the districts of Jeollabuk-do province might be more prone to avian influenza high case density due to nearby rivers and streams flowing from the Yellow Sea (Migratory stopover location). Sullivan et al. [51] stated that until most of the birds evacuated the Yellow Sea, which is known as a migratory stopping region, the bird species would be linked to outbreaks. Inland waterways, such as lakes and marshes used by waterfowl, were thought to be one of the most critical risk factors for disease propagation. If waterfowl harbor the HPAI virus, bodies of water might be a source of infection for other carriers such as resident birds, or rodents spreading the virus onto nearby farms. The presence of adjacent inland waters was considered to be a major determinant in the prevalence of HPAI in poultry [52]. When IAVs are kept in water under cold conditions, a near-neutral pH, and a low-to-moderate specific conductance, the viruses can survive and can stay infectious for months after adsorption [53].

As previously stated, Kernel density estimation indicated Chungcheongbuk-do province to have the greatest risk point prevalence rate. Chungcheongbuk-do is a province in central Korea that covers 70% of the country’s land, with the Sobaek mountainous region extending all along the province’s southern border [54]. It can be hypothesized that the environmental conditions, which are predominantly mountainous in the Chungcheongbuk-do province, may attract rodents and wild birds; this can pose an indirect risk of HPAI virus infection in chicken and duck farms. It is hypothesized that active surveillance of rodents and wild birds inhabiting these detected high-risk areas can be good practice. This may aid in identifying the key elements leading to future HPAI outbreaks, allowing HPAI occurrences to be managed to some extent.

There were certain limitations in our research analysis: Ripley’s K function is only applicable to symmetric point processes and does not allow for point weighting [55]. Although trying to disassociate these two effects (e.g., 1st order and 2nd order)—to truly comprehend the process leading to the observed events—is fundamental to the practice of spatial point process analysis, insinuating process form pattern is ultimately a matter of judgment and may not always be straightforward. Other limitations regarding our study included the following: After HPAI epidemics are reported, the nearby poultry farms within a range of 3 km are usually depopulated to prevent abrupt spread. Ducks do not show marked clinical signs, which make the early detection of HPAI infection difficult [28,35]. Moreover, the affected and non-affected regions’ poultry density and poultry depopulation ratio were not assessed. It is generally advised that the aforementioned factors be assessed using space–time interactions, to establish the spatial–temporal dynamics of the risk of HPAI H5NX viruses’ occurrence in the future.

## 7. Conclusions

The findings demonstrate a high density of infections within the affected areas or counties, as well as statistically significant clusters. They are also useful in determining the epidemic’s hot zones. Proactive efforts can be performed in the future to prevent disease outbreaks with the aid of the provided study.

## Figures and Tables

**Table 1:** Summary of five significant clusters identified in the HPAI H5N6 epidemic in South Korean poultry farms during 2016–2017. (**) represent the degree of level of significance.

Clusters	Cluster Centroid	Radius of the Cluster Window (km)	Total Population Cluster Window	Observed No. of Cases in Cluster Window	Expected No. of Cases in Cluster Window	Standardized Mortality Ratio	Risk Ratio	Proportion of Cases	Loglikelihood Ratio	*p* Value
Cluster 1	Eumseong, Chungcheongbuk-do	0.149	202	81	20.3926	3.97203	4.834	0.4009901	68.95386	0.001 **
Cluster 2	Cheonan, Chungcheongnam-do	0.147	104	46	10.49916	4.381303	4.876	0.4423077	42.3304	0.001 **
Cluster 3	Cheonan, Chungcheongnam-do	0.129	76	31	7.672462	4.040424	4.326	0.4078947	25.36896	0.001 **
Cluster 4	Jeongeup, Jeollabuk-do	0.0350	27	16	2.725743	5.869959	6.096	0.5925926	19.88819	0.001 **
Cluster 5	Pocheon-si, Gyeonggi-do	0.067	43	20	4.340998	4.607235	4.819	0.4651163	18.99753	0.001 **

Highest likelihood ratio was recorded for cluster 1 detected in Eumseong, Chungcheongbuk-do, followed by clusters 2 and 3 detected in Cheonan, Chungcheongnam-do. Likelihood ratio test was comparatively lower for cluster 4 detected in Jeongeup, Jeollabuk-do and cluster 5 detected in Pocheon-si, Gyeonggi-do.

## Data Availability

The dataset is available from the corresponding author upon reasonable request.

## Supplementary Materials

The following supporting information can be downloaded at: https://www.mdpi.com/article/10.3390/vetsci9030135/s1. Supplementary File.zip.
